# Monoclonal Antibodies as a Therapeutic Strategy against Multidrug-Resistant Bacterial Infections in a Post-COVID-19 Era

**DOI:** 10.3390/life14020246

**Published:** 2024-02-09

**Authors:** Hsiao-Chun Chen, Yu-Ling Pan, Ying Chen, Tsung-Hsuan Yang, Erh-Tung Hsu, Yu-Ting Huang, Ming-Hsien Chiang

**Affiliations:** 1 Department and Graduate Institute of Microbiology and Immunology, National Defense Medical Center, Taipei City 11490, Taiwan; 609070017@mail.ndmctsgh.edu.tw; 2 Department and Graduate Institute of Biology and Anatomy, National Defense Medical Center, Taipei City 11490, Taiwan; lab5303@mail.ndmctsgh.edu.tw (Y.-L.P.); ychen0523@mail.ndmctsgh.edu.tw (Y.C.); 3 School of Nursing, National Defense Medical Center, Taipei City 11490, Taiwan; 409040009@office365.ndmctsgh.edu.tw; 4 School of Medicine, National Defense Medical Center, Taipei City 11490, Taiwan; 408010143@mail.ndmctsgh.edu.tw (E.-T.H.); 408010086@mail.ndmctsgh.edu.tw (Y.-T.H.); 5 mProbe Taiwan Inc., Taipei City 105037, Taiwan

**Keywords:** antibiotic resistance, vaccine, monoclonal antibody, antibacterial infection

## Abstract

The development of severe multidrug-resistant bacterial infections has recently intensified because of the COVID-19 pandemic. According to the guidelines issued by the World Health Organization (WHO), routine antibiotic administration is not recommended for patients with supposed or confirmed mild SARS-CoV-2 infection or pneumonia, unless bacterial infection is clinically suspected. However, recent studies have pointed out that the proportion of non-essential antibiotic use in patients infected with SARS-CoV-2 remains high. Therefore, the silent pandemic of antibiotic resistance remains a pressing issue regardless of the present threats presented by the COVID-19 pandemic. To prevent or delay entry into the postulated post-antibiotic era, the long-term advocacy for the rational use of antibiotics, the optimization of infection control procedures, and the development of new antibacterial agents and vaccines should be underscored as vital practices of the antibacterial toolbox. Recently, the development of vaccines and monoclonal antibodies has gradually received attention following the advancement of biotechnology as well as enhanced drug discovery and development in cancer research. Although decent progress has been made in laboratory-based research and promising results have been obtained following clinical trials of some of these products, challenges still exist in their widespread clinical applications. This article describes the current advantages of antibacterial monoclonal antibodies, the development of associated clinical trials, and some perceived future perspectives and challenges. Further, we anticipate the development of more therapeutic agents to combat drug-resistant bacterial infections as well as to increase the resilience of current or novel agents/strategies.

## 1. Escalating Challenge of Antimicrobial Resistance in the Post-COVID Era

The global issue of antimicrobial resistance has been escalating rapidly. Each year, drug-resistant bacterial infections claim approximately 23,000 lives in the United States and 33,100 in Europe [1,2]. By 2050, the annual global death toll from multidrug-resistant (MDR) bacterial infections is projected to escalate to a staggering 10 million people [3]. A recent meta-study revealed that while nearly three-quarters of patients with SARS-CoV-2 were prescribed prophylactic antibiotics, only a mere 8.6% had confirmed bacterial co-infections (95% Confidence Interval 4.7–15.2%). This inappropriate and continuous antibiotic prescription practice significantly contributes to the rise in antibiotic resistance [4]. In the aftermath of the COVID-19 pandemic peak in 2020, the U.S. Centers for Disease Control and Prevention (CDC) reported a substantial increase in antibiotic use [5]. Consequently, the proportion of hospital-acquired antimicrobial-resistant microbial infections rose by 15%, including common pathogens such as MRSA (13%), VRE (14%), MDR *P. aeruginosa* (32%), and carbapenem-resistant *Acinetobacter* (78%) 5. These bacterial strains were responsible for 73.4% of all attributable deaths [6]. Despite the World Health Organization (WHO) and various national health agencies advocating for and establishing treatment guidelines during the COVID-19 pandemic, the overuse of antibiotics persists as a grave concern. This has intensified the silent pandemic of MDR bacteria [7], leading to increased mortality rates, prolonged hospital stays, and escalating medical costs. This poses a significant threat to both public health and national economies [1,8].

The urgency of infection control against antibiotic-resistant bacteria is evident in the post-antibiotic era [9]. As the problem of bacterial resistance continues to grow, the global population, especially in clinical settings, is becoming increasingly aware of various infection control measures. These include environmental cleaning and disinfection, hand hygiene, and the avoidance of inappropriate antibiotic use. However, the number of first-time drug approvals from 2020 to 2022 was disappointingly low and the rate of drug resistance growth has outpaced that of new drug discovery and development [10]. In light of this predicament, it is imperative to explore strategies beyond the development of new antibiotics. The development of immunotherapy, such as vaccine and monoclonal antibodies (mAbs), against MDR bacteria is one such promising strategy [11,12,13].

## 2. Developmental Challenges in Bacterial Vaccines

From the discovery and development of human medicines to the recent experience with COVID-19, vaccines have proven to be the most cost-effective strategies for the prevention of infectious diseases, even in immunocompromised populations [14]. Several studies have demonstrated that vaccinating against MDR bacteria is cost-effective, particularly in children aged 5 years or younger, as well as in lower middle-income and low-income countries where the burden of infectious diseases is relatively higher [15,16]. A recent study also estimated that pneumococcal conjugate vaccine (PCV13) reduced antibiotic-nonsusceptible invasive pneumococcal disease from 61% to 27% across all age groups in the U.S. [17]. Other studies have compellingly demonstrated typhoid conjugate vaccines (TCVs) as effective in reducing *Salmonella typhi* transmission in low-income countries. The World Health Organization (WHO) systematically confirms the TCV’s effectiveness in preventing typhoid fever spread in endemic regions, endorsing its inclusion in routine immunization programs, particularly in high-risk countries [18]. While antibiotics are gradually losing efficacy, there is a heightened urgency to develop new treatment strategies to combat the ever-changing MDR bacterial strains [19]. To highlight the urgency in the development of new therapeutic strategies, the focus on vaccines has shifted from being a mere topic of discussion to investigating their feasibility in clinical applications [20,21]. In multipopulational models, vaccination can inhibit resistance if it has a larger impact on subpopulations that consume more antibiotics [22]. However, three major challenges exist as hurdles in the development of MDR bacterial vaccines, viz., technical aspects, applicable groups, and economic considerations [23].

First, in technical identification of a suitable vaccine candidate are subunits from previous methods of utilizing virulence factors, surface sugar molecules, or capsules, as well as outer membrane proteins as antigens. Subsequently bioinformatics was employed to screen for proteins with epitope potential, conducted after the exposure of cell surface and highly conserved proteins. Moreover, translating findings from animal models to human clinical trials could be challenging due to variations in the expression levels of cytokines and differences in immunological checkpoints between humans and rodents [24]. Following these time-consuming and energy-intensive verification processes, very few successful candidates were identified [25,26].

Second, determining the applicable groups entails answering the question “what are the main target groups for vaccines?” Currently, only high-risk groups are targeted, such as patients in intensive care centers, patients with chronic diseases, those using ventilators, and individuals with cancer or undergoing surgical procedures. However, the protective effect during preoperative vaccination or at the onset of contracting diseases is limited. Therefore, defining high-risk groups in practice becomes challenging, potentially limiting the promotion of vaccines.

Last, the market for vaccines against MDR bacteria is currently not substantial. According to statistics from the United States (U.S.) Center for Disease Control and Prevention, approximately 2.8 million patients develop MDR bacterial infections every year [2]. Despite this large number, the incidence of developing such an infection is lower than that of other diseases; however, MDR bacterial infections still exhibit higher mortality rates. Moreover, the healthcare-related MDR bacterial infections have been proven to lead to an increase in the expensive healthcare expenses related to intensive care unit stays and prolonged hospital admissions [27]. Even so, in terms of economic benefits, biopharmaceutical companies are still encountering huge investment costs in R&D and considering short usage/profits lifespans, making this field less attractive when compared to the field of cancer treatment.

Therefore, in response to the current challenge of MDR bacterial infections, and considering the continuous development of new antibiotics in the race against drug resistance, a novel treatment approach such as the development of therapeutic mAbs might prove to be a more feasible strategy [23,28]. Unlike vaccines, which may take several weeks to induce protective immunity, administration of an mAb provides immediate protection. Thus, the purpose of this review is to provide a comprehensive overview of the development and application of mAbs in the treatment of MDR bacterial infections. This includes an examination of the mechanism of action of mAbs, a discussion of current research and clinical trials, and an analysis of the challenges and limitations associated with mAb treatment. Furthermore, this review aims to offer a comparative analysis of mAbs with other treatments for MDR bacterial infections and to discuss the regulatory and ethical considerations associated with their use. Ultimately, this review seeks to provide insights into the future perspectives of mAb treatment in the context of MDR bacterial infections, with the goal of guiding future research and development in this field.

## 3. Developmental Process of Antibacterial Monoclonal Antibodies

MAbs are homogenous antibodies derived from a single B-cell clone, capable of detecting a single epitope in an antigen. In the past, while numerous antibiotics were available on the market, the cost of using mAbs as treatment options was excessively high. Therefore, in comparison with the fields of cancers and autoimmune diseases [29], the development of antibacterial mAbs has progressed relatively slower [30]. Currently, with the advancement of precision medicine and biotechnology, there is a growing demand for mAbs in anti-infective clinical applications. In comparison with antibiotics, the application of mAbs as treatment strategies for bacterial infections offers several advantages: (1) high specificity, precisely combating MDR bacteria; (2) high safety profile, without harming normal intestinal flora [31]; (3) the potential for combination with regular antibiotics (antibody–drug conjugates), thereby reducing the dose and presenting with selective pressure [32]; (4) affinity and safety of mAbs that can potentially be enhanced through genetic engineering, such as single-chain fragment variable (scFv) antibodies and fully human antibodies [33,34]; (5) long half-life, ensuring bioavailability for several weeks to months after administration, theoretically providing dosing, compliance, and adherence benefits [31]; (6) therapeutic advantages for immunocompromised patients and those for whom vaccination is inappropriate [35]; (7) production with minimal chemical usage compared to antibiotics, promoting environmental friendliness [36]; (8) easy degradation under various conditions, including temperature changes, pH shifts, or oxidation, thus preventing accumulation in the environment like antibiotics [37]; and (9) drug resistance is less likely to occur because they target specific virulence factors rather than essential survival proteins [38]. Furthermore, in several clinical trials, mAbs have been explored as potential adjuncts to antibiotic therapy. MAbs have the capacity to deliver antibiotics directly to the site of infection, mitigating the excess toxicity and collateral damage associated with antibiotic use. This approach not only minimizes antibiotic-related side effects but also allows for the reduction in antibiotic dosages. By reducing antibiotic dosages, the selection pressure exerted by antibiotics could be diminished, thereby decreasing the likelihood of MDR development. While these endeavors hold promise for improving treatment outcomes, the ultimate goal continues to be the reduction or replacement of antibiotic usage [32,39,40,41].

**Figure 1:**
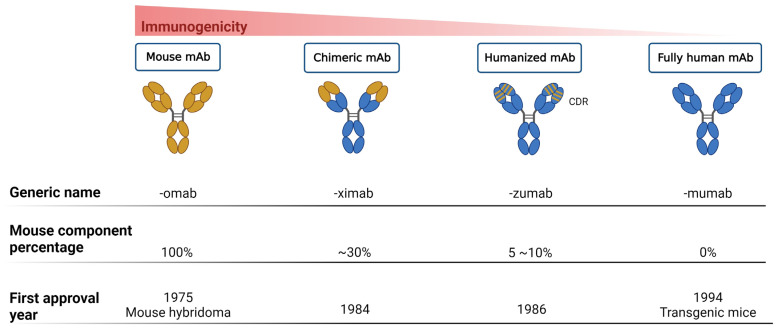
Progression of monoclonal antibody development. This figure illustrates the evolution of mAb technology, distinguishing between murine-derived antigens (indicated in yellow) and human-derived antigens (indicated in blue), to highlight the transition from animal-based to fully humanized antibody production for therapeutic use.

Thus far, mAbs can mainly be broadly categorized into mouse-derived, human–mouse chimera, humanized, and fully human mAbs, based on the type of development source [42] (Figure 1). The mouse-derived mAb is the hybridoma formed by the fusion of B lymphocytes of immunized mice and mouse myeloma cells. This represents the first generation of mAb preparation technology and is extensively used in antibody research. The human–mouse chimeric mAb is introduced into myeloma cells after genetic recombination between the variable region (Fv) gene on the mouse antibody and the constant region (Fc) of the human antibody, retaining approximately 30% of the murine antibody properties [34]. Humanized antibodies use the sequences of the complementarity determining regions (CDRs) in the mutant regions of murine mAbs to replace the corresponding positions in the variable regions of human antibodies, achieving approximately 90% humanization [43]. This type of antibody possesses the specificity of murine mAbs and retains affinity in humans. Fully human mAbs represent the most desirable option for mAb therapy. This category of mAb eliminates human heterogeneity across different species, thereby diminishing the risk of a human anti-chimeric antibody (HACA) response [33,34]. The preparation techniques for fully human mAbs mainly include the expression of the phage antibody library, ribosome display technology, and transgenic mouse technology [44]. In any case, mAbs are perceived as foreign antigens by the individual’s immune system, leading to the production of antibodies that can neutralize their effects or induce a pathological immune response. All chimeric mAbs inherently contain murine fragments, which inevitably produce HACA responses. While, humanized or fully human mAbs might elicit anti-drug antibody response, affecting pharmacokinetics (PKs) and mAb potency [31].

This figure delineates the evolution of mAbs, highlighting the reduction in immunogenicity from mouse to fully human mAbs. The upper bar graph illustrates the decreasing immunogenicity across four mAb generations: mouse, chimeric, humanized, and fully human mAbs. Below, three lines detail the generic name, mouse component percentage, and first approval year for each mAb type, showcasing the advancements in mAb development over time. 

## 4. Current Research and Clinical Trials

**Figure 2:**
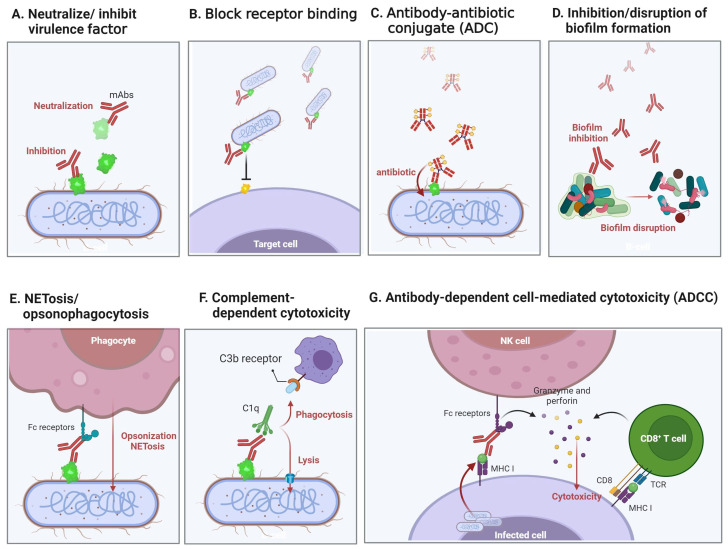
Multifaceted mechanisms of mAbs against bacterial infections. This figure illustrates the complex mechanisms through which mAbs counteract bacterial infections. (**A**) Highlights the neutralization or inhibition of bacterial virulence factors by mAbs, mitigating their pathogenic effects. (**B**) The process of mAbs blocking receptor-mediated adhesion is depicted, preventing bacterial adherence to host cells and hindering the progression of infection. (**C**) Portrays the Antibody–Antibiotic Conjugate (ADC) strategy, where mAbs conjugated with antibiotics enhance the precision and effectiveness of bacterial targeting and elimination. (**D**) Focuses on the role of mAbs in inhibiting or disrupting biofilm formation, a primary bacterial defense mechanism, facilitating bacterial clearance. (**E**) Illustrates the mAb-mediated NETosis/opsonophagocytosis pathway, promoting bacterial clearance through the facilitation of neutrophil extracellular traps (NETs) and enhanced phagocytosis. (**F**) Delineates the activation of complement-dependent cytotoxicity by mAbs, leading to bacterial cell lysis. (**G**) Illustrates the synergy between innate and adaptive immune responses facilitated by mAbs in Antibody-Dependent Cell-Mediated Cytotoxicity (ADCC), enhancing the clearance of bacterial infections [39,47,48,49].

Table 1 summarizes the development of clinical trials for antibacterial mAbs as recorded on the ClinicalTrials.gov website [45]. From the presented summary, it is evident that the main targets for the developed antibacterial mAbs are neutralizing toxins/virulence factors (bacterial exotoxins, perforin systems), highly conserved surface carbohydrates and outer membrane proteins, as well as biofilms and iron ion capture functional factors. Upon targeting these elements, the agents subsequently block host cell bacterial invasion, reduce biofilm formation, neutralize toxins, and induce complement, with consequent opsonophagocytosis and other immunomodulatory functions that subsequently occur in immune cells (Figure 2). Furthermore, since antibacterial mAbs are not directly bactericidal, but instead enhance the immune response against them or attenuate bacterial pathological activity, drug resistance due to selective pressure is less likely to occur [31,46].

In addition to Raxibacumab (GlaxoSmithKline, London, UK) and Obiltoxaximab (Elusys Therapeutics, Inc., Pine Brook, NJ, USA), which are anti-*Bacillus anthracis* toxins of strategic significance for bioterrorism, the most successful products that have undergone clinical trials were those for the treatment of repeated outbreaks of *Clostridium difficile* infection, including Bezlotoxumab (Zinplava) (Merck & Co., Rahway, NJ, USA) [50]. In 2016, the U.S. FDA approved its usage in high-risk population groups aged 18 and above, undergoing antibiotic treatment, including those on broad-spectrum antibiotics, enduring long-term use of gastric acid inhibitors, and individuals who have undergone gastrointestinal surgery [50]. Bezlotoxumab, a human IgG1 mAb, primarily targets toxin B of *C. difficile*, which neutralizes and reduces toxin damage to the intestinal wall and reduces inflammation. The successful experience from using mAbs in the treatment of *C. difficile* stimulated the continuous development of related antibacterial mAbs (Table 1). 

Furthermore, in addition to reducing drug resistance by minimizing selection pressures, mAbs can directly target essential outer proteins contributing to drug resistance, such as the type 3 secretion system (T3SS) in *P. aeruginosa* or outer membrane proteins in *A. baumannii* and *Salmonella* spp. [51,52]. Huang et al. demonstrated this in their study after the immunization with *A. baumannii* outer membrane vesicles. The production of polyclonal antibodies could significantly enhance the susceptibility of MDR *A. baumannii* to levofloxacin and ciprofloxacin in both in vivo and in vitro settings. This effect was attributed to their targeting of several outer membrane proteins [53]. These findings underscore the potential of mAbs as a formidable tool in combating infections caused by MDR bacteria.

In addition, recent advancements in mAbs have shifted toward a multivalent combinatorial model, including mAb cocktails with multiple epitope binding sites, which encompass the neutralizing antigen to a greater extent, closely resembling the human natural immune system [54,55]. A recent study proposes an engineered, multivalent protein biologic agent that targets five surface proteins and neutralizes five different *S. aureus* virulence factors [56]. It is designed to resist proteolysis, avoid Fc binding to *S. aureus* IgG-binding proteins, and neutralize toxins. The agent incorporates a pair of tandem centyrin moieties (small protein scaffolds derived from the fibronectin type III-binding domain) that bind to and neutralize two leukocidins of *S. aureus*, thereby protecting phagocytes and enhancing their antimicrobial function. Moreover, collecting antibodies from multiple B-cell lineages yielded varying site affinities for the same pathogen. The presence of multiple epitope binding sites provided a broader range of neutralization options and reduced the chance of pathogens developing escape mutations. This, in turn, inhibited or slowed down the development of MDR bacteria [57]. Multivalent antibacterial mAbs have the capacity to bind to numerous antigenic sites, which could fill the gap left by antibiotics and vaccines after treatment failure [58]. In summary, the combined use of mAbs alongside antibiotic therapy is a multi-attack mode approach that would build evolutionary barriers against bacteria and reduce the likelihood of treatment failure from the development of drug-resistant strains [48].

## 5. Novel Monoclonal Antibody Formats in Combatting Bacterial Infection

Antibody–drug conjugations, bispecific mAbs, IgYs, Nanobodies, and scFvs are novel types of mAbs that have gained significant attention in recent years (Table 2). Antibody–drug conjugation is a technique where drugs or toxins are covalently attached to the immunoglobulin [59]. Bispecific mAbs combine two distinct mAbs to simultaneously target two different proteins [60]. IgY is an immunoglobulin present in birds and reptiles [61]. Nanobodies are single-domain antigen-binding fragments derived from camelid heavy-chain antibodies [62]. ScFv is a fusion protein of the variable regions of the heavy and light chains of immunoglobulins, and single-domain antibody (sdAb) consists of either a light chain variable region or heavy chain variable region [63]. Each novel mAb type possesses unique characteristics and advantages. Antibody–drug conjugates can deliver drugs or toxins to specific sites, holding particular promise for killing cancer cells or microbes [59]. Bispecific mAbs can cause multiple physiological or anti-tumor responses by targeting two antigens or epitopes simultaneously [60]. IgY has potential applications in the treatment of *P. aeruginosa* and *S. aureus* infections [64,65]. Nanobodies offer advantages such as small size, high stability, and ease of production [62]. ScFv molecules have shown promise in treating neovascular age-related macular degeneration [66].

However, it is crucial to weigh the drawbacks of these novel mAb types as well. For instance, antibody–drug conjugates may encounter challenges related to drug stability and off-target effects [73]. Bispecific mAbs can be challenging to manufacture due to their complex structures [60]. IgY-based therapies may face issues related to immunogenicity and purification [61]. Nanobodies may have limitations in terms of tissue penetration and immunogenicity [62]. ScFv molecules can be prone to aggregation and have a shorter half-life compared to full-length antibodies [63]. In summary, novel mAb types such as antibody–drug conjugations, bispecific mAbs, IgYs, Nanobodies, and scFvs present distinct prospects for therapeutic interventions against MDR bacteria. Each type comes with its own set of advantages and disadvantages that warrant careful consideration. Further research and development efforts are needed to fully explore the potential of these novel mAb types in a post-COVID-19 era.

## 6. Shortcomings and Future Prospects of Antibacterial Monoclonal Antibodies

Although mAb-based immunotherapy is on the cusp of a booming research area in treating MDR bacterial infections (Table 1), several developmental challenges remain. First, despite the use of humanized mAbs, there might still be a chance of developing an HACA response, leading to therapeutic risks [29]. Second, mAb targets are typically specific to the antigens of particular bacteria; therefore, the rapid and immediate diagnosis of pathogens will be, to a greater extent, very important. In addition, in some cases, the target antigen may only be expressed in a specific circulating strain, a single organ infection, or a disease period, which might limit the effectiveness of mAbs. For example, KB001-A targeting the T3SS protein PcrV of *P*. *aeruginosa* successfully reduced the incidence of pneumonia caused by *P. aeruginosa* infection in patients using ventilators. However, it did not demonstrate the same efficacy in alleviating infection in patients with cystic fibrosis [74]. Furthermore, highly conserved outer membrane proteins, while suitable as targets, are often masked by bacterial cell surface carbohydrates, making them less accessible to mAbs. Second, the variability in exopolysaccharide structures across serotypes poses a challenge, as a single mAb may not effectively target all variations [75]. For example, *Streptococcus pneumoniae*, a common cause of pneumonia and meningitis, produces a capsule composed of exopolysaccharides. This capsule is crucial for the bacterium’s virulence and its ability to evade the host’s immune system [76]. The composition and structure of this exopolysaccharide capsule vary among different serotypes of *S. pneumoniae*. This variation in exopolysaccharide structures among serotypes implies that a single mAb might not provide broad protection against all serotypes of the bacterium. In addition, PNAG (β-1-6-linked poly-N-acetyl-d-glucosamine), a highly conserved exopolysaccharide in at least 75 pathogens, including Gram-positive and Gram-negative bacteria, molds, and parasites, was related to survival, toxicity, and biofilm formation of pathogens [77]. Owing to this, a specific IgG1 mAb (F598) was developed using deacetylated synthetic PNAG as an antigen due to its ability to mediate the killing of PNAG-expressing microbial pathogens. Despite the demonstrated efficacy of mAb F598 in mitigating microbial challenges across various models and microbes [77,78], its Phase 2 clinical trials were not pursued further. However, given its promising results in earlier phases, it holds potential for future developments. Last, similar to the problems observed for vaccines and antibiotics, commercial investment tends to be influenced by market size of the disease. Therefore, the development of broad-spectrum mAbs needs to be guided by the policies of government regulators to reduce commercial barriers and enable related fields to blossom. This remains an open question that requires addressing in the foreseeable future.

## 7. Conclusions

In conclusion, with the advancement in the development of mAbs, screening, production, and engineering technologies need to be streamlined to gain a deeper understanding of the performance. A comprehensive understanding of target antigens in disease pathogenesis prior to pathogen-specific mAbs being formally introduced onto the therapeutic candidate list is needed. MAbs exert their antibacterial effects through a variety of mechanisms, including opsonophagocytosis, complement-mediated bactericidal activity, inhibition of biofilm formation, and neutralization of bacterial toxins. These pharmacodynamic mechanisms differ from those of small-molecule antibacterial agents, and the high specificity of mAbs could reduce interference with the normal flora, thus reducing selection pressure for cross-resistance. Therefore, when paired with appropriate infection control measures, mAbs could allow for more flexible treatment strategies against drug-resistant bacterial strains. Despite the abovementioned advancements, certain hurdles in this field must be overcome, necessitating focused research and development efforts to focus on establishing clinically relevant in vitro assays and animal models to enhance the correlation of preclinical and clinical findings. Although only three mAbs for the prevention or treatment of bacterial infections are currently available in the market, given the numerous advantages of these agents over traditional antibacterial agents, as well as positive findings is some clinical studies, research on broad-spectrum antibacterial mAbs is expected to progress rapidly in the future.

## Figures and Tables

**Table 1:** The ongoing clinical trials for antibacterial mAbs are accessible on ClinicalTrials.gov (accessed on 1 August 2023).

Agents	Bacterial Species	mAb Target	Sponsor (s)	Phase of Trial	NCT Number	Origin
Tefibazumab	*Staphylococcus aureus*	clumping factor A	Bristol Myers Squibb	II	NCT00198289	Humanized
514G3		cell wall moiety Protein A (SpA)	XBiotech	II	NCT02357966	Human (isolated and cloned from a healthy human donor)
MEDI4893 (Suvratoxumab)		alpha-hemolysin	Medimmune	II	NCT02296320	Human (VelocImmune mice)
ASN-100 (ASN-1 and ASN-2)		alpha-hemolysin, gamma-hemolysin, bicomponent leucocidin (HlgAB, HlgCB, LukED, LukSF, and LukGH)	Arsansis	II	NCT02940626	Human
AR-301 (Tosatoxumab)		alpha toxin	Aridis Pharmaceuticals	III	NCT03816956	Human (convalescent patient B-cell)
DSTA-4637S		Teichoic acid (Antibody–Antibiotic Conjugate)	Genentech and Roche	I	NCT03162250	Human
Aurograb^®^		ABC transporter GrfA	NeuTec Pharma/Novartis	III	NCT00217841	scFv
KB001	*Pseudomonas aeruginosa*	type III secretion system, PcrV	KaloBios	II	NCT00638365	Humanized PEGylated Fab
PsAer-IgY		surface protein (Flagellin)	Mukoviszidose Institut gGmbH	III	NCT01455675	Chicken egg yolk
AR-105 (Aerucin)		alginate	Aridis Pharmaceuticals	II	NCT03027609	Human
KBPA-101 (Aermab)		LPS O-antigen (serotype O11)	Aridis (Kenta Biotech)	II	NCT00851435	Human
MEDI3902		type III secretion system, PcrV, exopolysaccharide, Psl	Medimmune	II	NCT02696902	Human (bispecific phage display and VelocImmune mouse)
MK-3415A (actoxumab-bezlotoxumab)	*Clostridium difficile*	toxin A/B	Merck Sharp & Dohme	III	NCT01513239	Human
Bezlotoxumab (Zinplava^®^)		toxin B	Merck Sharp & Dohme	IV	NCT03880539	Human
GS-CDA1/MDX-1388		toxin A/C-terminal toxin B fragment	MassBiologics/Merck	II	NCT00350298	human
Raxibacumab (ABthrax^®^/Anthrin^®^)	*Bacillus anthracis*	protective antigen (PA) component of anthrax toxin	Human Genome Sciences	IV	NCT02177721	Human (phage display)
Obiltoxaximab (Anthim^®^, ETI-204)		PA component of anthrax toxin	Elusys Therapeutics	IV	NCT03088111	Human–mouse (hybridoma)
MDX-1303 (Valortim^®^)		uncleaved and cleaved PA	PharmAthene	I	NCT00964561	Human
AVP-21D9 (ThravixaTM)		PA component of anthrax toxin	Emergent BioSolutions	I	NCT01202695	Human
NTM-1632/3	*Clostridium botulinum*	botulinum neurotoxin type B	NIAID	I	NCT02779140	Humanized
XOMA 3ab		botulinum neurotoxin type B	XOMA/NIAID	I	NCT01357213	Humanized
TRL1068	Biofilm—multiple species	biofilm scaffolding proteins DNABII	Trellis Bioscience	I	NCT04763759	Human
F598	Multiple species	poly-N-acetylglucosamine (PNAG)	Alopexx Pharmaceuticals	II	NCT03222401	Human
Pagibaximab (BSYX-A110)	*Staphylococcal Sepsis*	lipoteichoic acid	Biosynexus	III	NCT00646399	Humanized
cαStx1/cαStx2	Shiga Toxin-Producing *E. coli*	shiga toxins	Thallion Pharmaceuticals	II	NCT01252199	Humanized

**Table 2:** The novel types of mAbs.

Novel mAb Types		Characteristics	Examples	Advantages	Disadvantages	Refs.
Antibody–Drug conjugation	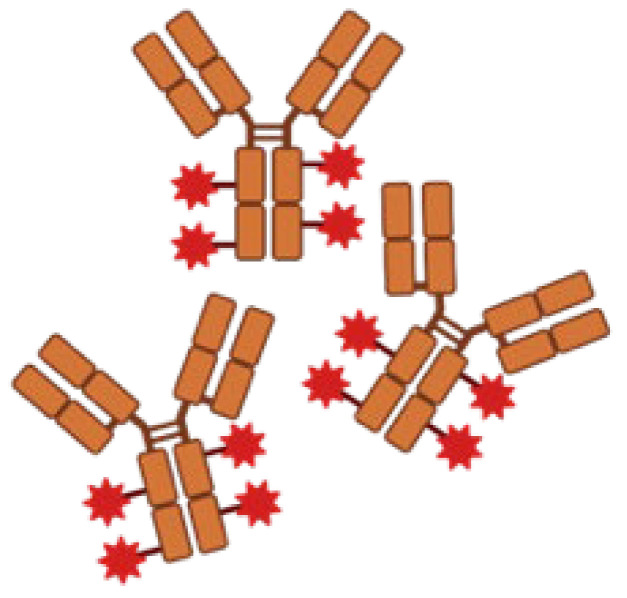	Delivering drugs directly to specific antigens.	DSTA4637A/MRSA/Human IgG1-dmDNA31	Potentially minimized the side effect of antibiotics. Stable in circulation. Revive antibacterials that show poor in pharmacokinetic properties or undesired host toxicity.	The amount of antibiotics is resticted by the abundance of the antigen on bacterial cell surfaces. Composition of various components with distinct chemical characteristics. Challenging in manufacturing.	[32,39]
Bispecific mAbs	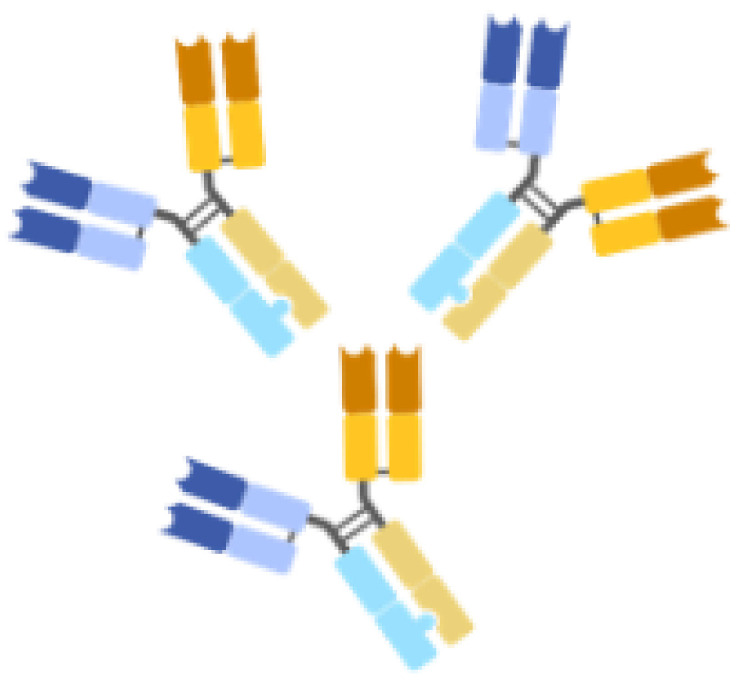	Simultaneously binds to two different epitopes using an mAb.	MEDI3902/*P. aeruginosa* PcrV and Psl	An alternative therapeutic approach substituting combina-tion therapy with two monospecific drugs. The sensitive immuno-assays enable swift and straightforward detection of infectious diseases.	Production by two separate cell lines makes it costly and challenging to harvest. Decreased stability or a susceptibility to aggregation.	[60,67,68]
IgY	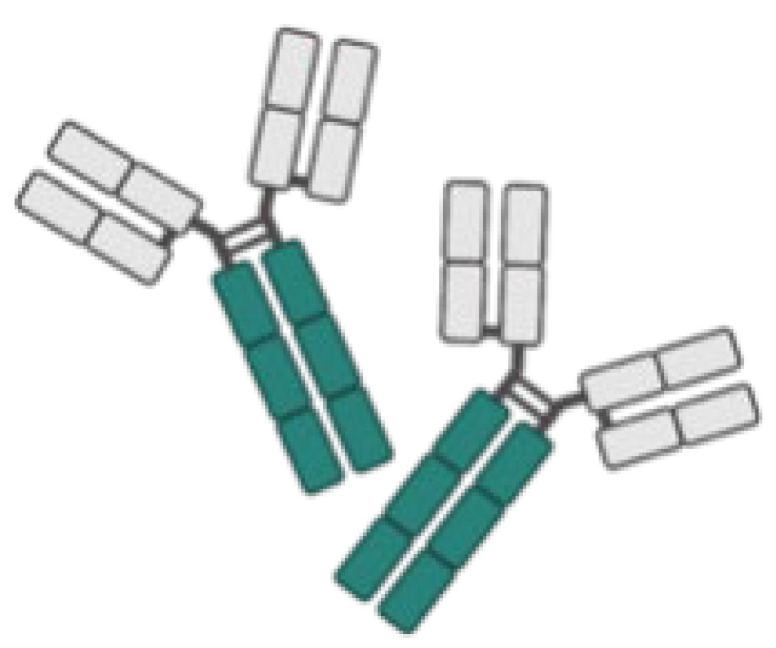	Highly conserved to human IgG.	PsAer-IgY/*P. aeruginosa* surface protein (Flagellin)	Low-cost and minimal production effort renders. Rapid production is feasible for acute illnesses. Lower background of cross-reactivity in diagnostic assays.	The possibility of host-produced anti-IgY antibodies. Unable to activate human Fc receptors and complement system.	[64,69]
Nanobody	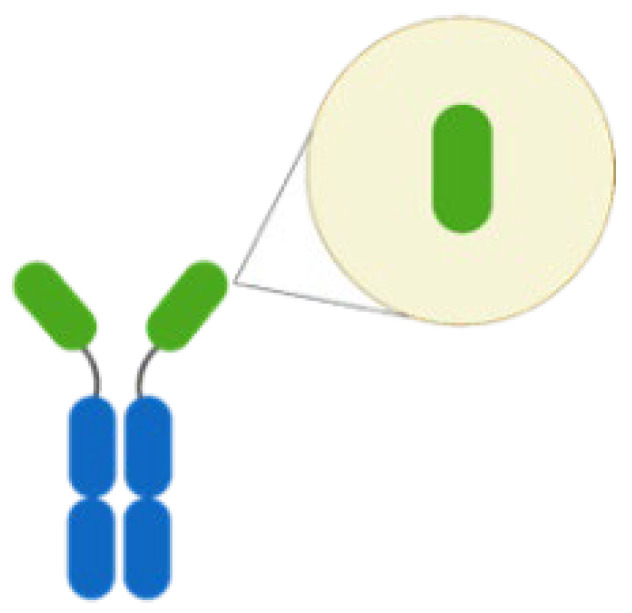	Antibody lack of light chain and constant domain.	NbD7/Ehrlichia chaffeensis translocated factor-1	Identify recesses or concealed epitopes inaccessible to mAbs. Demonstrate remarkable stability, hydrophilicity, and solubility, contributing to their sustained binding capacity. Diverse expression systems.	High uptake in kidney. Risk of immunogenecity.	[62,70]
scFv	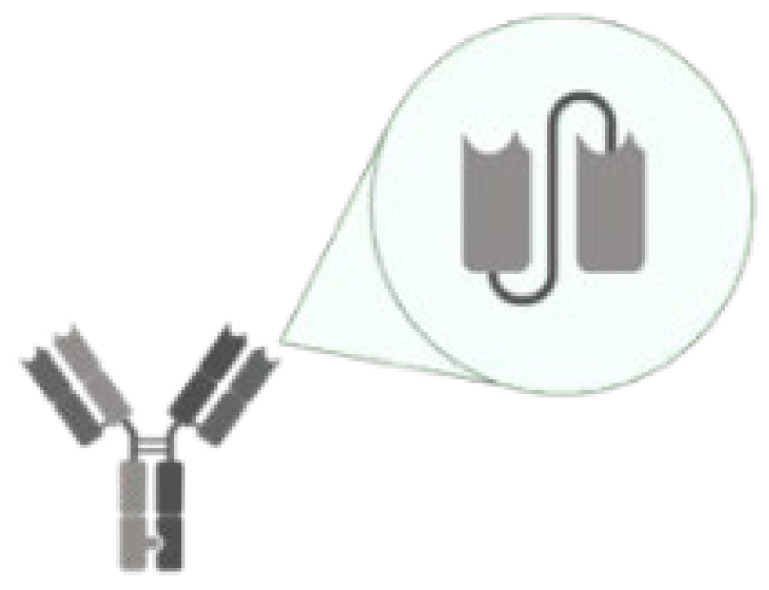	An antibody comprises only the variable regions of the heavy and light chains.	Brolucizumab/Neovascular Age-related Macular Degeneration VEGF-A	Preserve the original antibody’s binding affinity and specificity. Easily constructed, expressed, and manufactured in large quantities.	Limited half-life and low stability in circulation. Requiring high doses and continuous administration in in vivo applications.	[66,71,72]

## Data Availability

Data sharing is not applicable.
